# Personalized downstaging treatment with ADT, chemotherapy and add-on zimberelimab for very-high-risk clinically localized prostate cancer: A case report

**DOI:** 10.1097/MD.0000000000032870

**Published:** 2023-02-10

**Authors:** Jie Li, Tengfei Gu, Shengping Hu, Liang Wang, Ting Chen

**Affiliations:** a Department of Urology, Lishui Central Hospital and Fifth Affiliated Hospital of Wenzhou Medical College, Lishui, China; b Medical Affairs Department, Guangzhou Gloria Biosciences Co. Ltd., Beijing, China.

**Keywords:** downstaging therapy, high-risk, PD-1, prostate cancer, zimberelimab

## Abstract

Very-high-risk prostate cancer (PCa) is associated with poor prognosis. Radical prostatectomy (RP) is an option for selected high-risk PCa cases, especially in younger, healthier patients. However, a high Gleason score and high T stage can increase the risk of RP. Neoadjuvant therapy has been reported in high- or very-high-risk PCa, but its clinical use remains controversial.

**Diagnoses and patient concerns:**

A 53-year-old male patient diagnosed with PCa was referred to our hospital. The patient’s Gleason score was 4 + 5, and the clinical stage was T4N0M0, with an abnormally enlarged prostate adhering to the rectum and leading to decreased mobility of the rectum, suggesting a very-high-risk PCa inappropriate for RP. However, instead of external beam radiation therapy, which is the standard treatment for inoperable PCa, the patient insisted on RP.

**Interventions:**

Androgen deprivation therapy plus docetaxel was chosen as the first downstaging treatment; however, the tumor was too slightly downsized to undergo RP. Therefore, zimberelimab was added after confirmation of a genomic feature of high microsatellite instability and high tumor mutational burden status.

**Outcomes:**

After 4 doses of zimberelimab, the prostate shrank significantly. The patient successfully completed RP after another dose of zimberelimab, and achieved a pathological complete response (pCR).

**Lessons:**

Our case represents a successful attempt at personalized treatment and provides preliminary evidence for the clinical use of downstaging therapy of androgen deprivation therapy, chemotherapy, and add-on zimberelimab for very-high-risk clinically localized PCa.

## 1. Introduction

Prostate cancer (PCa) is the second most common cancer in men, with 1,414,259 new cases and 375,304 new deaths worldwide in 2020.^[1]^ The national comprehensive cancer network guidelines for prostate cancer stratify patients into different risk groups based on clinical/pathologic features. High-risk PCa was defined as cT3a, Grade Group 4/5, or prostate-specific antigen (PSA) > 20ng/mL. For patients with cT3b-cT4, primary Gleason pattern 5(Gleason Score 9 or 10), 2 or 3 high-risk features included or > 4 cores with Grade Group 4/5, the risk group will be stratified to very high.^[2]^ High-risk features of PCa are related to poor prognosis, with an increased risk of cancer-related death compared to low- or intermediate-risk disease.^[3,4]^

Treatment based on external beam radiation therapy or radical prostatectomy (RP) is recommended for high- or very-high-risk groups. However, because of potential perioperative morbidity, RP is only suitable for patients with a life expectancy of > 10 years. According to the national comprehensive cancer network guidelines, RP is an option for patients with high-risk disease and in selected patients with very-high-risk disease, whose disease is expected to be completely excised surgically and has no serious comorbidity, especially for younger, healthier patients.^[2]^ However, RP alone is often inadequate for high- or very-high-risk patients, and a high Gleason score or T stage may increase the risk of RP. Previous data showed that neoadjuvant therapy with androgen deprivation therapy (ADT) alone or combined with chemotherapy can help downsize tumors, reduce surgery risk, decrease rates of positive surgical margins, and eliminate micro-metastasis.^[5,6]^ However, the clinical use of neoadjuvant therapy remains controversial.

Therefore, for clinically high- or very-high-risk patients, personalized therapy that considers both patient preference and clinical characteristics is of great importance. Precision medicine, which involves tailoring customized treatment regimens to individual patients, has been used clinically. Since different individuals possess unique characteristics at genomic, biochemical, and behavioral levels, different strategies need to be applied accordingly, especially when targeted therapy or immunotherapy has evolved.^[7,8]^

Here, personalized downstaging treatment for a newly diagnosed very-high-risk T4 locally advanced PCa patient is described. The patient was considered inappropriate for RP at the time of diagnosis. external beam radiation therapy was usually considered as first choice for similar patients; however, since he was young and had a strong willingness to pursue RP, downstaging therapy with docetaxel plus ADT was applied. Zimberelimab was added due to the weak effect of initial therapy after 2 crucial programmed cell death-1 (PD-1) therapy biomarkers, high microsatellite instability (MSI-H) and high tumor mutational burden (TMB-H)^[9]^ genome features, were confirmed. Downsizing on imaging before RP was achieved, and a pathological complete response was confirmed with RP tissue. This case provides evidence of the effectiveness of personalized downstaging therapy with ADT, chemotherapy, and add-on zimberelimab in patients with very-high-risk prostate cancer.

## 2. Case presentation

**Figure 1.**
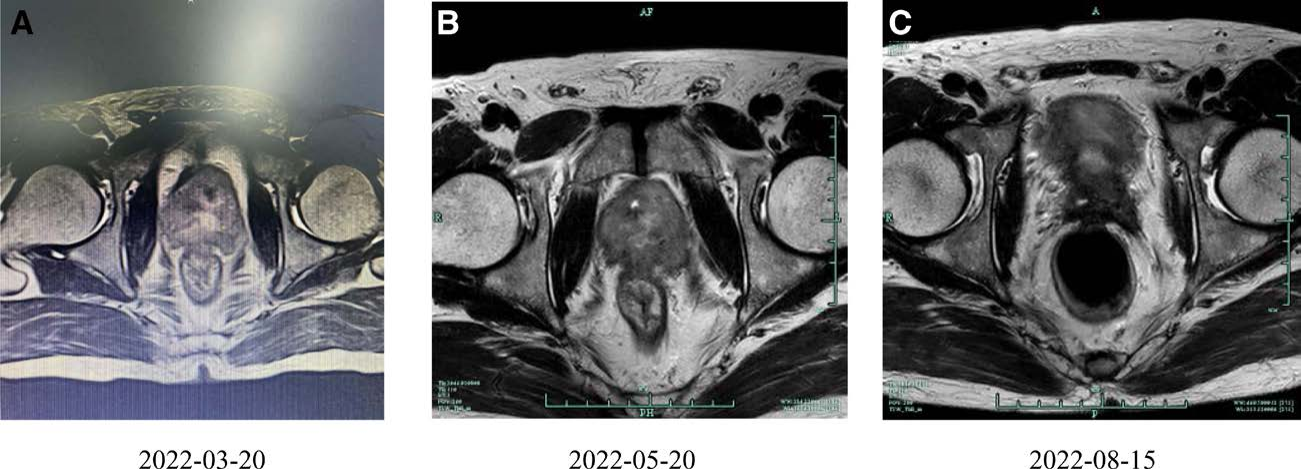
MRI of prostate disease at baseline and course of treatment. A)MRI results of baseline on 2022-03-20. Size of prostate: 5.3*6.0*5.7cm B) MRI results after 2 courses of bicalutamide and 3 courses of docetaxel on 2022-05-20. Size of prostate: 4.7*4.5*5.2cm. C) MRI results after 4 courses of zimberelimab on 2022-08-15. Size of prostate: 2.8*3.0*3.6cm. MRI = magnetic resonance imaging.

**Figure 2.**
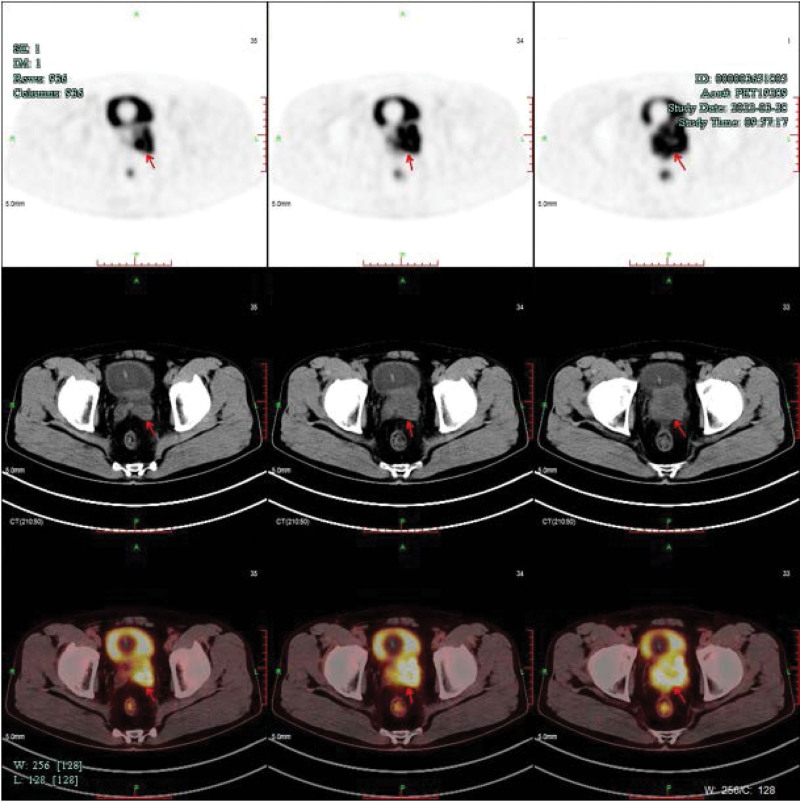
PET-CT of prostate disease at baseline on 2022-03-28. PET-CT = positron emission tomography-computed tomography.

A 53-year-old male patient diagnosed with prostate cancer for 4 days was referred to our hospital in March 2022. Level of PSA, free PSA and testosterone was 0.75ng/mL, 0.2ng/mL, and 6.48ng/mL, respectively. Digital rectal examination revealed an abnormally enlarged prostate adhering to the rectum, leading to decreased rectal mobility. Size of prostate was 5.3*6.0*5.7 cm measured by magnetic resonance imaging (MRI) (Fig. 1A). Transrectal prostate needle biopsy performed on March 23^th^ showed a Gleason score of 4 + 5 adenocarcinoma in both lobes. Positron emission tomography-computed tomography confirmed invasion of the seminal vesicle, compressing the rectum and bladder, but no distant metastasis (Fig. 2). The final clinical staging was T4N0M0, reflecting very-high-risk PCa. The patient was also classified as inoperable considering the T4 tumor, a high Gleason score, and invasion of the surrounding organ.

However, at an early age and in good health, the patient was strongly willing to pursue RP. After evaluation, neoadjuvant therapy was commenced on March 30^th^ for down-staging and enhancing the success rate of RP, including docetaxel (100 mg, IV gtt, Q3W), goserelin (3.6 mg, ih, Q4W), and bicalutamide (50 mg, PO, qd).

Immunohistochemistry and next-generation sequencing of paraffin sections of primary prostate lesions were performed and the results were received on April 9^th^. PD-L1 was negatively expressed measured using the clone 22C3 pharmDx kit (Agilent Technologies, Inc., Santa Clara, CA), but results of next-generation sequencing revealed MSI-H and TMB-H (TMB = 64.87 mutations/Mb) status in the genome, indicating a potential sensitivity to anti-PD-1 therapy. However, the anti-PD-1 antibody was not included in the initial therapy because of the high cost of the drug.

**Figure 3.**
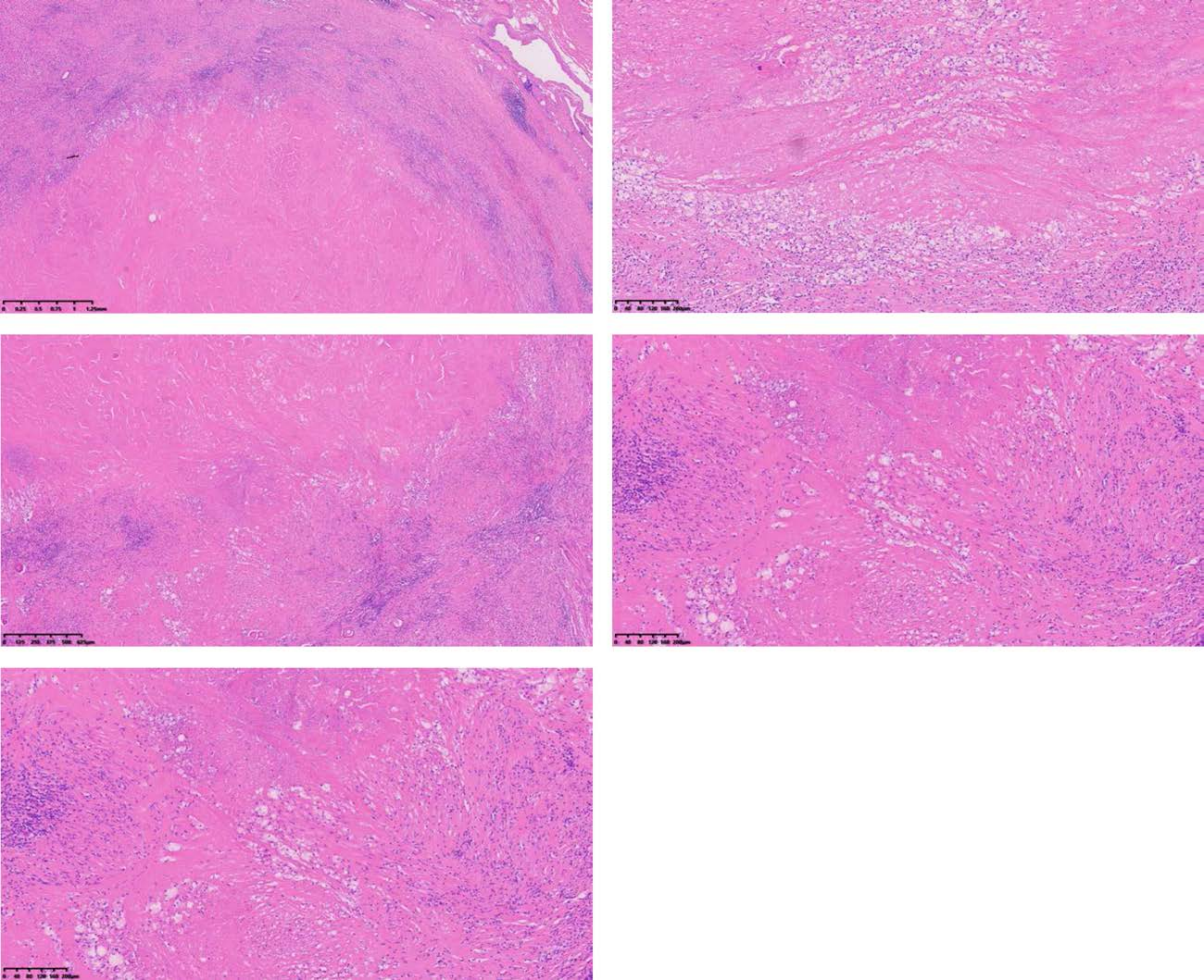
Pathological complete response of RP tissue. RP = radical prostatectomy.

After 2 courses of bicalutamide and 3 courses of docetaxel, size of prostate shrunk to 4.7*4.5*5.2 cm on May 30^th^, determined by MRI (Fig. 1B). The disease burden was partly relieved after neoadjuvant docetaxel and ADT; however, the prostate had not reached the proper size for RP surgery and was still conglutinating to the rectum. Therefore, to facilitate the recession of the prostate, considering the TMB-H and MSI-H genome features of the patient, we added zimberelimab to the neoadjuvant agents after discussion with the patient and his relatives. The dose of zimberelimab was 120 mg Q3W, and after the last dose of docetaxel was completed on July 14^th^, zimberelimab was continued with ADT. After 4 courses of zimberelimab, the prostate shrunk significantly, and conglutination to the rectum was alleviated. MRI showed a prostate size of 2.8*3.0*3.6 cm on August 15^th^, and results of digital rectal examination suggest a remarkable improvement of local condition, meeting the requirement for RP surgery (Fig.1C). To further reduce the risk of rectal injury, an additional dose of zimberelimab was administered on August 22^nd^. On September 8^th^, the patient was transferred to a tertiary cancer hospital and underwent a robot-assisted radical prostatectomy. The operation was successful, and surgical pathology revealed no remaining tumor cells, achieving a pathological complete response (Fig. 3).

## 3. Discussion

High-risk PCa, including very-high-risk PCa patients, had an increased chance of developing biochemical recurrence, disease progression, metastases, and death compared to low-risk or intermediate-risk PCa. Prostate cancer-specific mortality rates for high-risk PCa are 28.8% and 35.5% at 10 and 15 years, respectively.^[10]^ Standard treatment for this population remains controversial. Published data show that high-risk PCa patients can benefit from RP. RP for clinical T4 PCa increased survival compared with radiation therapy or hormone therapy alone and was comparable to radiation therapy plus hormone therapy.^[11]^ The European association of urology (EAU) guidelines recommend RP as a reasonable option for high-risk PCa patients with tumors of low volume and not fixed to the pelvic wall or invasion of the urethral sphincter.^[10]^

In our case, the patient was diagnosed with very-high-risk PCa, with a Gleason score of 4 + 5 in both lobes and clinical stage T4N0M0. The patient’s prostate was abnormally enlarged and adhered to rectum, leading to decreased mobility of the rectum. These features suggest an increased risk for RP surgery, and the patient seemed to be more prone to clinical regression and metastases, indicating an inoperable status and poor prognosis. However, to better debulk the tumor and enhance local control of the disease, the patient insisted on undergoing RP. In addition to physicians’ clinical experiences, patients’ values, needs, and preferences are also important for improving patient outcomes. Therefore, patient involvement is needed when making decisions about treatment regimens.^[12]^ In our circumstances, downstaging treatment before surgery may be helpful.

Clinical data support therapy before surgery for downsizing tumors, reducing surgery risk, regaining operability for selected non-operable diseases, and eliminating micro-metastasis in many malignant tumors. Conventional ADT is the most common agent for neoadjuvant therapy, since PCa is largely androgen-driven, and androgen receptor is widely expressed throughout the course of the disease.^[13]^ Neoadjuvant ADT therapy prior to RP for high-risk PCa has been proven to reduce positive margin rates, tumor volume, and lymph node invasion, but long-term benefits have not been observed, such as 5-year biochemical recurrence rate and cancer-related death.^[13,14]^ The feasibility and preliminary efficiency of neoadjuvant chemotherapy plus ADT has also been proven. Neoadjuvant chemohormonal therapy has been shown to improve biochemical progression-free survival, metastasis-free survival, and overall survival compared to RP alone, but the routine use of neoadjuvant chemohormonal therapy is still controversial.^[15]^ For this patient, after fully understanding the patient’s demands and informing them of the potential risks, ADT plus docetaxel was chosen as first downstaging treatment. However, the initial treatment downsized the tumor too slightly to allow RP, so other agents were needed.

With the emergence of next-generation sequencing, knowledge of cancer has deepened, and clinicians have begun to realize the differences between different patients in terms of physical factors such as age, performance status, and medicine tolerance, as well as molecular features such as DNA mutation, RNA, or protein expression. Using clinical tools to better understand the disease and choosing a specific treatment for certain patients is thought to be effective and crucial.^[8]^ Immune checkpoint inhibitors, such as anti-PD-1 antibodies, and have been widely used to treat various cancers. However, prostate cancer is known to be insensitive to immunotherapy, and the clinical activity of anti-PD-1 antibodies in unselected PCa is modest.^[16]^ To select patients who can benefit from immunotherapy, individualized treatment has been practiced for metastatic PCa, especially metastatic castration-resistant prostate cancer. MSI and TMB are the 2 most important biomarkers for the precision treatment of PD-1 therapy, and anti-PD-1 antibody pembrolizumab has been proven by FDA for MSI-H or TMB-H solid tumors.^[17,18]^ Considering that the patient was MSI-H and TMB-H in the genome, which supports the potential response to anti-PD-1 therapy, we finally added zimberelimab to downstaging chemohormonal therapy. The prostate size decreased significantly after 4 courses of zimberelimab. The patient finally completed RP successfully and achieved pathological complete response. To our knowledge, this is the first case of personalized downstaging therapy of chemohormonal combined with an add-on PD-1 inhibitor in very-high-risk clinically localized PCa. The outstanding response on both radiography and pathology provides preliminary evidence for the clinical use of downstaging therapy in similar patients and represents a successful attempt at personalized treatment.

## Author contributions

**Data curation:** Tengfei Gu, Shengping Hu.

**Investigation:** Jie Li.

**Funding acquisition:** Ting Chen.

**Supervision:** Ting Chen.

**Writing – original draft:** Liang Wang.

**Writing – review & editing:** Jie Li.

## References

[1] Sung H Ferlay J Siegel RL. Global cancer statistics 2020: GLOBOCAN estimates of incidence and mortality worldwide for 36 cancers in 185 countries. CA Cancer J Clin. 2021;71:209–49. 33538338 10.3322/caac.21660

[2] Schaeffer EM Srinivas S. NCCN Clinical Practice Guidelines in Oncology-Prostate Cancer (Version 1.2023 – September 16, 2022). http://www.nccn.org/.

[3] Akre O Garmo H Adolfsson J. Mortality among men with locally advanced prostate cancer managed with noncurative intent: a nationwide study in PCBaSe Sweden. Eur Urol. 2011;60:554–63. 21664039 10.1016/j.eururo.2011.05.047

[4] Rider JR Sandin F Andrén O. Long-term outcomes among noncuratively treated men according to prostate cancer risk category in a nationwide, population-based study. Eur Urol. 2013;63:88–96. 22902040 10.1016/j.eururo.2012.08.001

[5] Tafuri A Cerruto MA Antonelli A. Neoadjuvant strategies before radical prostatectomy for high risk prostate cancer in the era of new hormonal agents. Curr Drug Targets. 2021;22:68–76. 32564752 10.2174/1389450121666200621194409

[6] Zhuang J Zhang S Qiu X. Platinum-based neoadjuvant chemotherapy before radical prostatectomy for locally advanced prostate cancer with homologous recombination deficiency: a case report. Front Oncol. 2022;11:777318. 35070981 10.3389/fonc.2021.777318 PMC8766302

[7] Tsimberidou AM Fountzilas E Nikanjam M. Review of precision cancer medicine: evolution of the treatment paradigm. Cancer Treat Rev. 2020;86:102019. 32251926 10.1016/j.ctrv.2020.102019 PMC7272286

[8] Goetz LH Schork NJ. Personalized medicine: motivation, challenges, and progress. Fertil Steril. 2018;109:952–63. 29935653 10.1016/j.fertnstert.2018.05.006 PMC6366451

[9] Barata P Agarwal N Nussenzveig R. Clinical activity of pembrolizumab in metastatic prostate cancer with microsatellite instability high (MSI-H) detected by circulating tumor DNA. J Immunother Cancer. 2020;8:e001065. 32788235 10.1136/jitc-2020-001065 PMC7422632

[10] Mottet N Cornford P van den Bergh RCN. EAU - EANM - ESTRO - ESUR - SIOG Guidelines on Prostate Cancer. 2020. http://uroweb.org/guidelines/compilations-of-all-guidelines/.

[11] Johnstone PA Ward KC Goodman M. Radical prostatectomy for clinical T4 prostate cancer. Cancer. 2006;106:2603–9. 16700037 10.1002/cncr.21926

[12] Wang D Liu C Zhang X. Do physicians’ attitudes towards patient-centered communication promote physicians’ intention and behavior of involving patients in medical decisions? Int J Environ Res Public Health. 2020;17:63936393. 10.3390/ijerph17176393 PMC7503802 32887364

[13] Devos G Devlies W De Meerleer G. Neoadjuvant hormonal therapy before radical prostatectomy in high-risk prostate cancer. Nat Rev Urol. 2021;18:739–62. 34526701 10.1038/s41585-021-00514-9

[14] Wang X Zhang J Han B. Neoadjuvant hormonal therapy for prostate cancer: morphologic features and predictive parameters of therapy response. Adv Anat Pathol. 2022;29:252–8. 35670702 10.1097/PAP.0000000000000347

[15] Eastham JA Heller G Halabi S. Cancer and leukemia group B 90203 (Alliance): radical prostatectomy with or without neoadjuvant chemohormonal therapy in localized, high-risk prostate cancer. J Clin Oncol. 2020;38:3042–50. 32706639 10.1200/JCO.20.00315 PMC7479762

[16] Antonarakis ES Piulats JM Gross-Goupil M. Pembrolizumab for treatment-refractory metastatic castration-resistant prostate cancer: multicohort, open-label phase II KEYNOTE-199 study. J Clin Oncol. 2020;38:395–405. 31774688 10.1200/JCO.19.01638 PMC7186583

[17] U.S. Food and Drug Administration. FDA grants accelerated approval to pembrolizumab for first tissue/site agnostic indication. Available at: https://www.fda.gov/drugs/resources-information-approved-drugs/fda-grants-accelerated-approval-pembrolizumab-first-tissuesite-agnostic-indication.

[18] U.S. Food and Drug Administration. FDA approves pembrolizumab for adults and children with TMB-H solid tumors. Available at: https://www.fda.gov/drugs/drug-approvals-and-databases/fda-approves-pembrolizumab-adults-and-children-tmb-h-solid-tumors.

